# Application of Solid-Phase Extraction and High-Performance Liquid Chromatography with Fluorescence Detection to Analyze Eleven Bisphenols in Amniotic Fluid Samples Collected during Amniocentesis

**DOI:** 10.3390/ijerph19042309

**Published:** 2022-02-17

**Authors:** Tomasz Tuzimski, Szymon Szubartowski

**Affiliations:** 1 Department of Physical Chemistry, Medical University of Lublin, Chodźki 4a, 20-093 Lublin, Poland; szymon.szubartowski95@gmail.com; 2 Doctoral School of Medical University of Lublin, Medical University of Lublin, Chodźki 7, 20-093 Lublin, Poland

**Keywords:** bisphenols, amniotic fluid samples, fluorescence detector (FLD), solid-phase extraction (SPE), Scherzo SM-C18, amniocentesis

## Abstract

Amniocentesis involves taking a sample of the amniotic fluid in order to perform a karyotype test and diagnose any genetic defects that may affect the fetus. Amniotic fluid has been collected from patients with an indication for amniocentesis in the 15–26th week of pregnancy. A simple and sensitive high-performance liquid chromatography with fluorescence detection (HPLC-FLD) method for identification and quantification of eleven selected bisphenols in amniotic fluid samples is proposed. The proposed method involved protein precipitation using acetonitrile, and next the extraction and concentration of analytes by solid-phase extraction (SPE). The solid-phase extraction (SPE) procedure with application of Oasis HLB SPE columns performed well for the majority of the analytes, with recoveries in the range of 67–121% and relative standard deviations (RSD%) less than 16%. The limits of detection (LODs) and quantification (LOQs) of all the investigated analytes were in the range of 0.8–2.5 ng mL^−1^ and 2.4–7.5 ng mL^−1^ (curves constructed in methanol) and 1.1–5.2 ng mL^−1^ and 3.2–15.6 ng mL^−1^ (curves constructed in the amniotic fluid), respectively. The method was validated at the following two concentration levels: 10 ng mL^−1^ (2 × LOQ) and 20 ng mL^−1^ (4 LOQ). The results confirm the validity of the SPE procedure and HPLC-FLD method for identification and quantification of bisphenols in amniotic fluid samples collected during an amniocentesis. The result obtained show that HPLC-FLD is a useful method for determination of bisphenol residues at nanogram per milliliter concentrations in amniotic fluid samples. Residues of five analytes (BADGE·2H_2_O, BPAF, BADGE, BADGE·H_2_O·HCl and BADGE·2HCl) were detected in amniotic fluid samples. Additionally, the harmfulness of bisphenols as potential pathogens that may cause karyotype disorders and contribute to preterm birth was estimated.

## 1. Introduction

Worldwide, the annual production of plastics has reached 8300 million metric tons, with ~80% accumulating in the environment, many of which considered to contain endocrine-disrupting chemicals [1,2]. Bisphenols are a widely used plastic compound with endocrine-disrupting properties that ubiquitously affect the endocrine system [3]. Bisphenol A (BPA) binds estrogen receptors such as estrogen-related receptor α (ERα), estrogen-related receptor β (Erβ), membrane estrogen receptor (mERα) and estrogen-related receptor γ (ERRγ), which might upregulate the mRNA expression of endothelial nitric oxide synthase by activating one of the estrogenic receptors, ERα [4]. BPA is used in the manufacture of polycarbonate plastic and epoxy resins and is found in different products such as food packaging (e.g., cans for drinks and foods), pet food packaging, personal care products, dental sealants, thermal receipts, cans for storing paints and varnishes, and other [5,6]. Diet is considered an important source of bisphenols. Certain food groups such as canned food, fish, meat and poultry have been associated with bisphenol levels. Due to its toxicity, BPA has been banned in the manufacture of infant feeding bottles and toys in Europe since 2011 [7]. Due to BPA toxicity, the *European Food Safety Authority* (EFSA) evaluated scientific studies and set the maximum specific migration limit (SML) level to 0.05 milligrams per kilogram of food (mg/kg) in 2018, updating its previous level set in 2011 [8]. Consequently, bisphenol analogues, such as BPS and BPF, are used as a substitution for BPA in some consumer products. However, bisphenol analogues also exhibit toxic effects, such as endocrine disruption, fertility problems, neurotoxicity and cytotoxicity. One of the recent studies reported that the most common BPA substitutes, such as bisphenol S (BPS), bisphenol F (BPF), bisphenol AF (BPAF), bisphenol B (BPB), bisphenol AP (BPAP), bisphenol E (BPE) and bisphenol Z (BPZ), have agonistic effects on ERα and Erβ, where BPAF was the strongest agonist. Furthermore, apart from binding with ERα/β receptors, BPA analogues can also bind with androgen receptor (AR), glucocorticoid receptor (GR), pregnane X receptor (PXR) and constitutive androstane receptor (CAR) [4]. As the fluorinated homologue of BPA, a proven endocrine-disrupting compound, there is concern that bisphenol AF (BPAF) is potentially more harmful to human health because its CF_3_ moiety may be much more electronegative and reactive than the CH_3_ of BPA. The acute oral toxicity of BPAF in laboratory animals is low [9], but recent research indicates that this chemical may pose high risk as a potential endocrine disruptor to humans and wildlife via binding with hormone receptors. In vitro assays indicate that bisphenol AF (BPAF) binds to estrogen receptor-alpha approximately 20 times more effectively than BPA and to estrogen receptor-beta almost 50 times more effectively [9].

After oral ingestion, BPA suffers a metabolic process and is excreted mainly as BPA-glucuronide in urine with a half-life of less than 6 h. The metabolism of bisphenol analogues (BPS and BPF) and excretion in urine is less known, but it seems to be similar to BPA. Therefore, BPA, BPF and BPS have already been included as prioritized substances to be determined in human biomonitoring (HBM) studies [10]. Several biomonitoring studies have been implemented in order to determine the urinary levels of bisphenols, especially BPF and BPS. Therefore, urine is the most suitable matrix for monitoring the content of such bisphenols. Due to the low concentrations of free BPA and its conjugated substances in the urine matrix, such as glucuronide and disulfate as chlorides forms, sample preparation often includes hydrolysis of the BPA–glucuronide and BPA–disulfate conjugate, clean-up and pre-concentration [10].

Children are particularly susceptible to environmental exposures compared to adults. Firstly, children consume more food, water and air per pound of body weight compared to adults and therefore are proportionally exposed to more environmental toxicants [11]. Moreover, an individual’s susceptibility to environmental chemical exposure is greatest during the specific time periods when complex organs, pathways and connections are being established; i.e., during prenatal and early life development [12]. Exposure to environmental chemicals during these periods can impact cell signaling and alter development.

In the past, despite appropriate sensitivity and detectability for trace analysis, the accuracy and precision of LC-MS was often limited by the presence of interferences from the sample matrix, especially for biological and environmental complex samples, such as urine. Matrix effects may occur when endogenous components compete with the analyte for available charges during the ionization process, leading to ion suppression or enhancement. Sometimes during LC-MS analysis, lipids and protein macromolecules can limit analyte transfer to the gas phase whilst other non-volatile substances, usually at higher concentrations, may cause ion suppression by a combination of physiochemical mechanisms, such as analyte co-precipitation, elevation of the solution boiling point and changes in the surface tension of the spray droplets. Despite certain limitations, tandem mass spectrometry with the possibility of using modern analyzers enables usually the quantitative analysis of bisphenols at the level of 0.1 ng mL^−1^ of sample.

High-performance liquid chromatography combined with a fluorescence detector (HPLC-FLD) can be applied to analysis of very small concentrations of analytes in biological samples. The FLD detector has the advantage of being sensitive to the identification of analytes in picograms per milliliter of sample (pg mL^−1^) [13]. Determination of such small amounts can be performed with the FLD detector thanks to the amplification of the analyte signal (ranging from 1 to 18) [13,14].

The diagnostic value of amniotic fluid (AF) is broad and has not yet been fully explored for prenatal diagnosis of pregnancies at risk from xenobiotics, environmental exposures and for the elucidation of mechanisms underlying important public health challenges, including on preterm birth. Despite significant progress in diagnosis, preterm delivery rates remain high. The assessment of human fetal exposure to chemicals is key to fully understand developmental toxicity. So far, only a few research results have been published on the identification of bisphenols (especially BPA) in the amniotic fluid samples [15,16].

In the present work, a low cost, specificity and sensitive method is proposed for identification and quantitative analysis of eleven bisphenols in amniotic fluid samples collected during an amniocentesis. To the best of our knowledge, this method is the first to combine the advantages of SPE as extraction techniques with HPLC-FLD for the identification and quantification of analytes in human amniotic fluid samples, which have been collected from patients with an indication for amniocentesis in the 15–26th week of pregnancy. Additionally, an attempt was made to correlate of concentrations of bisphenols with the possibility of karyotype disorders and preterm birth.

## 2. Materials and Methods

### 2.1. Bisphenols’ Standards and Their Purity

All standards’ purity was of ‘analytical-reagent grade’ and declared by the manufacturers as having purity, for all of the reference standards of bisphenols, ≥98.0%. The following standards used for the bisphenols under investigation were obtained from Sigma-Aldrich (Bellefonte, PA, USA): 3-[4-[2-[4-(2.3-Dihydroxypropoxy)phenyl]propan-2-yl]phenoxy]propane-1.2-diol (BADGE·2H_2_O), 4,4′-Methylenediphenol (bisphenol F—BPF), 1,1-Bis(4-hydroxyphenyl)ethane (bisphenol E—BPE), 2-[4-(3-Chloro-2-hydroxypropyloxy)phenyl]-2-[4-(2,3-dihydroxypropyloxy)phenyl]propane (BADGE·H_2_O·HCl), 3-[4-[2-[4-(Oxiran-2-ylmethoxy)phenyl]propan-2-yl]phenoxy]propane-1.2-diol (BADGE·H_2_O), 2,2-Bis(4-hydroxyphenyl)butane (bisphenol B—BPB), 2,2-Bis(4-hydroxyphenyl)hexafluoropropane (BPAF), 4,4′-(1-Phenylethylidene)bisphenol (BPAP), 1-Chloro-3-[4-[2-[4-(3-chloro-2-hydroxypropoxy)-phenyl]propan-2-yl]phenoxy]propan-2-ol (BADGE·2HCl), 2-[[4-[2-[4-(Oxiran-2-ylmethoxy)phenyl]propan-2yl]phenoxy]methyl]oxirane (BADGE) and 4,4′-(1,4-Phenylenediisopropylidene) bisphenol (BPP).

### 2.2. Solvents Applied as Mobile-Phase Composition and during SPE Experiments

Methanol (MeOH) was purchased from E. Merck (Darmstadt, Germany) and its purity indicated by the manufacturer was ‘hyper-grade for LC-MS’ analysis. Purities of other solvents used such as acetonitrile (MeCN), tetrahydrofuran (THF), n-heptane and formic acid (HCOOH) were of the ‘gradient grade for liquid chromatography’, and solvents were purchased also from E. Merck (Darmstadt, Germany). LC-MS grade water was purchased from Sigma-Aldrich (St. Louis, MO, USA). Deionized water (0.07–0.09 mS cm^−1^) was produced in our laboratory using a Hydrolab System (Gdańsk, Poland). All analytical equipment, including solvents and reagents, was checked for bisphenol contamination prior to all experiments (both SPE and HPLC analysis). Individual stock standard solutions were prepared in methanol and stored in screw-capped glass tubes in a refrigerator (+2 to +4 °C in the dark). Bisphenol standards were divided into two groups providing satisfactory separation: Mixture 1 (BADGE·2H_2_O; BPE; BADGE·H_2_O; BPAF; BADGE) and Mixture 2 (BPF; BADGE·H_2_O·HCl; BPB; BPAP; BADGE·2HCl; BPP). Both mixtures were prepared by diluting previously weighted analytes in methanol in glass tubes. These mixtures were used for calibration preparation as well as for the spiking of the amniotic fluid samples. All mixtures prepared during experiments were stored in glass tubes in the fridge under the same conditions for up to two weeks.

### 2.3. HPLC-FLD

An Agilent Technologies 1200 HPLC system was used consisting in a quaternary pump (G1311A), an autosampler (1260 Infinity II Vialsampler), thermostat of column (G1316A), and a fluorescence detector (Agilent Technologies 1260 FLD), which were used during the chromatographic analysis. The samples were thermostated by an autosampler at 8 °C.

### 2.4. HPLC-FLD Conditions Used during Analysis

Analytes were separated using a Scherzo SM-C18 (150 mm × 4.6 mm column with a 3-µm particle size, Agilent Technologies, Wilmington, DE, USA). The column was thermostated at 22 °C. The optimum mobile phase consisted of 50 mM HCOOH in water (component A) and 50 mM HCOOH in acetonitrile (component B) in a gradient elution: 0–10 min from 40% eluent B to 85% B; 10–16.5 min isocratic 85% B. The mobile-phase flow rate was 0.4 mL/min.

In order to elute interferences of the matrix, before the next step of human amniotic fluid sample analysis, the isocratic elution with 100% B as the mobile phase was applied for 15 min with a flow rate of 1 mL/min, and the next isocratic elution had the initial conditions.

FLD detection was carried out simultaneously at four different excitation wavelengths (225, 230, 235 and 240 nm). The emission wavelength was set at 300 nm.

During all chromatographic experiments with application of a fluorescence detector a moderate reinforcement (14) of studied bisphenols from the applicable reinforcement range (1–18) was used.

### 2.5. Method Validation

A validation study was performed using spiked human amniotic fluid samples and included evaluation of the selectivity, linearity, limits of detection (LODs), quantification (LOQs), matrix effects (ME), extraction recovery precision and accuracy.

#### 2.5.1. Selectivity

The selectivity was evaluated by analyzing the amniotic fluid samples from different sources to investigate potential interferences with the signals of the analytes. The extent of interferences originating from endogenous amniotic fluid sample components at the specific retention time of each analyte was evaluated through a comparison of an average blank amniotic fluid matrix sample (collected from ten women and then mixed) with the spiked average blank amniotic fluid matrix sample. HPLC analyses of bisphenol standards were repeated three times. The identification of bisphenols was accomplished on the basis of the retention times of the analytes simultaneously at four different optimal excitation wavelengths by a fluorescence detector.

#### 2.5.2. Linearity

Calibration curves for the LOD and LOQ values were constructed by analyzing the bisphenol standards divided into mixtures (please see Section 2.2) in methanol at six concentrations, from 0.5 to 20 ng mL^−1^, using six replicates. The calibration curves were obtained by means of the least-squares method.

The limits of detection (LODs) and limits of quantification (LOQs) obtained for bisphenols were calculated according to the formulas LOD = 3.3 (SD/S) and LOQ = 10 (SD/S), where SD is the standard deviation of the response (peak area) and S is the slope of the calibration curve. HPLC analyses of the bisphenol standards were repeated three times.

The identification of bisphenols was accomplished on the basis of the retention times of the analytes.

#### 2.5.3. Calculation of Relative Standard Deviation Values (RSD) and Average Extraction Recovery

**Figure 1:**
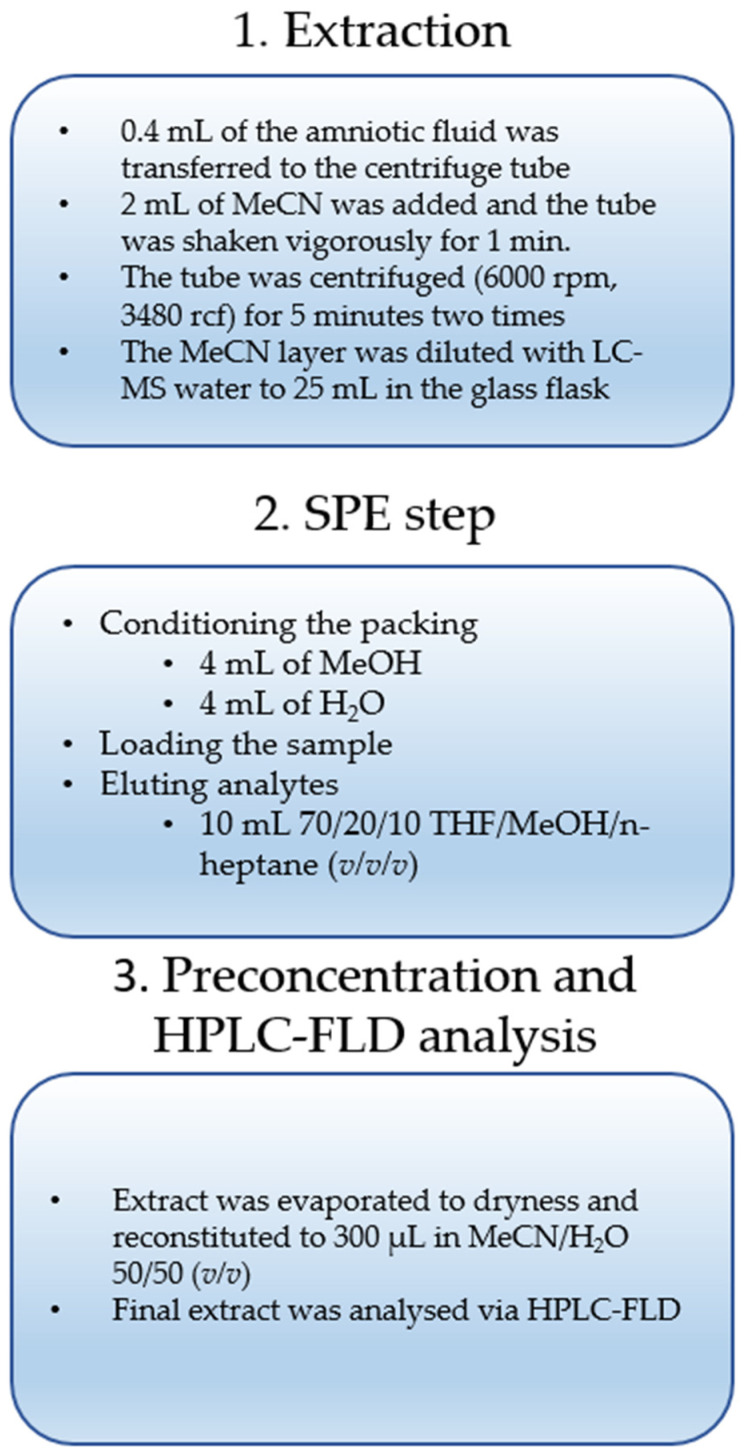
Flowchart of the optimized SPE-based extraction procedure applied for the detection of the bisphenols’ residues in human amniotic fluid samples.

For recovery studies, LOQ was estimated at the 5 ng mL^−1^ level according to results obtained during the linearity study described in the previous section. Average extraction recovery was evaluated at two concentration levels of 10 ng mL^−1^ (2 × LOQ) and 20 ng mL^−1^ (4 × LOQ), according to the following formula:

$$\mathrm{Recovery}\%\;=\frac{\mathrm{Average}\;\mathrm{analyte}\;\mathrm{concentration}\;\mathrm{found}\;\mathrm{in}\;\mathrm{the}\;\mathrm{spiked}\;\mathrm{human}\;\mathrm{amniotic}\;\mathrm{fluid}\;\mathrm{sample}}{\mathrm{Analyte}\;\mathrm{concentration}\;\mathrm{added}\;\mathrm{to}\;\mathrm{the}\;\mathrm{spiked}\;\mathrm{human}\;\mathrm{amniotic}\;\mathrm{fluid}\;\mathrm{sample}}\times 100\%$$

where the average analyte concentration found in the spiked human amniotic fluid sample is the peak area of the determined analyte in the sample before the procedure (explanation regarding spiking sample: the proper concentration of bisphenol was obtained after adding a solution of the standard to the average blank human amniotic fluid matrix sample before starting the procedure shown in Figure 1); and the analyte concentration added to the spiked human amniotic fluid sample is the peak area of the determined analyte in the post-extraction sample (explanation regarding the post-extraction sample: the proper concentration of bisphenol was obtained after adding a solution of the standard to the final extract of the average blank human amniotic fluid matrix sample after the SPE step and before evaporation of the final extract).

The relative standard deviation (RSD%) values were calculated as follows:

$$\mathrm{RSD}\%\;=\frac{\mathrm{Standard}\;\mathrm{deviation}\;\mathrm{of}\;\mathrm{the}\;\mathrm{recovery}\;\left(\%\right)}{\mathrm{Mean}\;\mathrm{recovery}\;\left(\%\right)}\times 100\%$$

#### 2.5.4. Matrix Effect—Assessment of the Degree of Matrix Interference

The matrix effect values were calculated as follows:

$$ME\%=\frac{x-y}{y}\times 100\%$$

*x*—peak area of analyte in matrix;
*y*—peak area of analyte in standard mixture.

The matrix effect values of the analytes were determined by constructing calibration curves in the ranges from 2.5 ng mL^−1^ to 50 ng mL^−1^ in the averaged matrix of amniotic fluid. In order to prepare an averaged amniotic fluid matrix, samples were taken from all patients and then mixed to ensure homogeneity.

### 2.6. Optimization of the SPE-Based Extraction Procedure

Amniotic fluid samples (0.4 mL) were transferred to 15 mL falcon centrifuge tubes and spiked with an appropriate amount of a mixture of bisphenol (1 or 2) standards and 2 mL of acetonitrile (MeCN) was added. Tubes were shaken vigorously for two minutes and centrifuged for 5 min, three times (6000 rpm, 3480 rcf).

After centrifugation the MeCN layer was transferred into a 25 mL glass flask and diluted to 25 mL of LC-MS water to prepare the sample for the SPE clean-up step. An Oasis HLB cartridge (400 mg sorbent per cartridge, 60 µm, Waters Corporation, Milford, MA, USA) was conditioned with 5 mL of methanol and 5 mL of water. Then, 25 mL of the sample was loaded. Then, analytes were eluted with 10 mL 1% HCOOH in 20/10/70 tetrahydrofuran (THF)/n-heptane/methanol (*v*/*v*/*v*).

The eluted solution was evaporated to dryness and reconstituted in 300 µL acetonitrile/water 50: 50 (*v*/*v*).

### 2.7. Human Amniotic Fluid Sample Collection

The samples have been obtained from the Department of Obstetrics and Pathology of Pregnancy, Medical University of Lublin, Poland (continuation of cooperation with Prof. Anna Kwaśniewska). Amniocentesis involves taking a sample of the amniotic fluid in order to perform a karyotype test and diagnose any genetic defects that may affect the fetus. Amniotic fluid has been collected in patients with an indication for amniocentesis in the 15–26th week of pregnancy. During amniocentesis, 23 mL of amniotic fluid was withdrawn, 20 mL of which was donated to genetic testing. The remaining 3 mL of amniotic fluid was the target sample for our study. Therefore, the method of collected/obtaining has not been an additional burden for women with an indication for amniocentesis. The object of the analysis was the aqueous fluid, which were microbiologically tested before the analysis. All samples were collected in glass bottles and immediately analyzed or frozen immediately at −23 °C until analysis.

Sample collection was conducted from July 2021 to September 2021. Amniotic fluid was collected in bisphenol-free tubes. All samples were collected and immediately analyzed or frozen at –23 °C until analysis.

This study was approved by the Bioethics Committee at the Medical University of Lublin, Poland (Resolution of the Bioethics Committee at the Medical University of Lublin No. KE-0254/239/2021).

#### Description of Patients Who Have Undergone Amniocentesis

The indication for amniocentesis was confirmation or exclusion of genetic aberrations, such as trisomy 13 (Patau’s syndrome), trisomy 18 (Edwards syndrome) and trisomy 21 (Down syndrome), and on heritable diseases in high-risk pregnancies, and also possibility of preterm birth.

The study group consisted of 20 women in high-risk pregnancy who were qualified for amniocentesis (Table 1). The inclusion criteria for the amniocentesis included the following: abnormal ultrasound image of the fetus characteristic for trisomy 13, 18 and 21, especially revealing as heart defects, generalized swelling of the fetus, spina bifida, megacystis, brain defect, hydronephrosis, risk of infections as well as family history of trisomy 13, 18, 21.

Four of the twenty women had pregnancy complications (fetal death during previous pregnancy). Newborns in these patients were born earlier and the presence of some bisphenols in the amniotic fluid samples was initially identified.

## 3. Results

**Figure 2:**
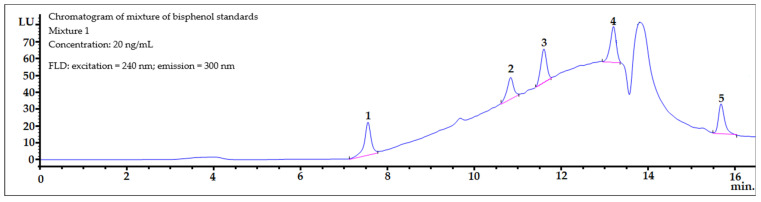

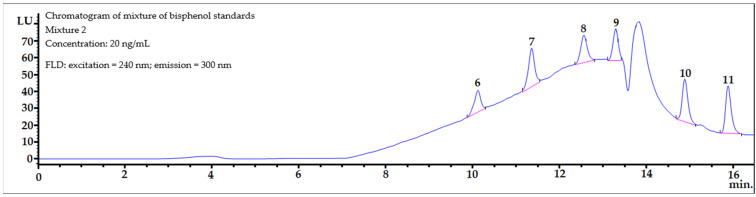
FLD chromatograms of Mixtures 1 (**top**) and 2 (**bottom**) of the bisphenol standards (20 ng mL^−1^).

The chromatographic condition described in the experimental section yielded satisfactory separation for the mixtures of 11 bisphenols studied, with total analysis time of less than 16 min (Figure 2). Logarithm of octanol-water partition coefficient values (Log *P*) for the 11 bisphenols selected in our experiments were from 2.1 for BADGE∙2H_2_O to 6.1 for BPP (Table 2). A wide polarity range of analytes is important to ensure that proposed extraction procedure (Figure 1) will cope with the broad range of bisphenols.

Results obtained from the validated studies in Table 3 demonstrated the good performance of the method, showing good linearity in the studied range (from 0.5 to 20 ng mL^−1^, using six replicates), good precision and adequate values of limits of detection (LODs) and quantification (LOQs).

Amniotic fluid (AF) is uniquely suited as a matrix for early detection of the association between fetal exposures and preterm birth due to its fetal origin and the fact that it is sampled from women who are at higher risk of preterm birth. It is not yet known whether EDCs, including bisphenol A (BPA) and other bisphenols, can affect the expression of proteins considered viable or potential biomarkers, e.g., for the onset of preterm birth.

The limits of quantitation (LOQs) were determined through the analysis of samples with known analyte concentrations and by establishing the minimum level at which an analyte could be quantified with acceptable levels of accuracy and precision [17,18].

The LOQ values of the analytes were determined by constructing calibration curves in the ranges from 2.5 ng mL^−1^ to 50 ng mL^−1^ in the averaged matrix of amniotic fluid (Table 4). In order to prepare an averaged amniotic fluid matrix, samples were taken from all patients and then mixed to ensure homogeneity. The calibration curves of the bisphenols under investigation showed satisfactory levels of linearity and a correlation between the concentration and peak area for the studied range with a determination coefficient of R^2^ ≥ 0.9888 (Table 4).

Analysis of biological samples requires good extraction techniques for sample preparation. In the current study, the authors proposed a rapid, efficient and reliable method for extraction of 11 bisphenols (BADGE·2H_2_O; BPE; BADGE·H_2_O; BPAF; BADGE; BPF; BADGE·H_2_O·HCl; BPB; BPAP; BADGE·2HCl; BPP) from amniotic fluid collected from patients with an indication for amniocentesis in the 15–26th week of pregnancy. The flowchart of the procedure is presented in Figure 1. At the beginning of the procedure, before the solid-phase extraction (SPE), the authors used acetonitrile extraction, which allowed for proper purification of the samples from protein interferences of the matrix. SPE can be used in amniotic fluid analysis, owing to the fact that it provides high concentration ratios. Solid-phase extraction (SPE) also enables satisfactory cleanup of ‘dirty’ samples. Bisphenols were enriched from amniotic fluid samples by solid-phase extraction on Oasis HLB SPE columns. The analytes were eluted with 10 mL 1% HCOOH in 20/10/70 tetrahydrofuran (THF)/n-heptane/methanol (*v*/*v*/*v*). The supernatant was evaporated to dryness under a fume hood. Afterwards, the remaining residues were reconstituted in 300 µL acetonitrile/water 50:50 (*v*/*v*).

**Figure 3:**
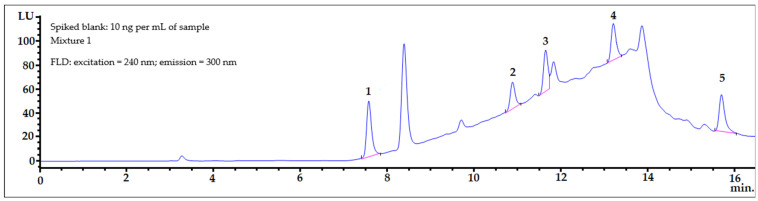

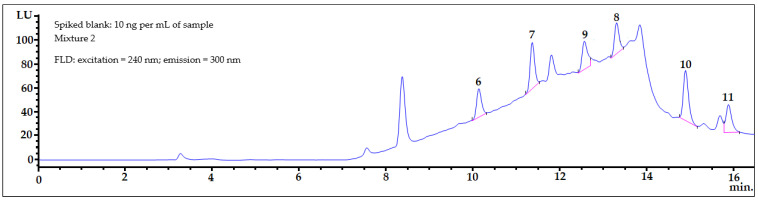
FLD chromatograms of spiked two blank human amniotic fluid matrix samples, which were spiked with Mixture 1 (**top**) and Mixture 2 (**bottom**) of bisphenol standards at the same level (20 ng mL^−1^). After the SPE procedure, the eluates were evaporated to dryness separately, and then separately reconstituted into 300 µL of mixture MeOH:H_2_O (50:50, *v*/*v*), and next both analyzed by HPLC-FLD.

Samples of amniotic fluid were spiked with the bisphenols under investigation at two concentrations levels of 10 ng mL^−1^ (2 × LOQ) and 20 ng mL^−1^ (4 × LOQ). Typical chromatograms of the spiked amniotic fluid samples are shown in Figure 3. The sample selected was a mixture of amniotic fluid collected from ten women during amniocentesis. This blank human amniotic fluid matrix sample was spiked with Mixture 1 (Figure 3, top) and Mixture 2 (Figure 3, bottom) of the bisphenol standards at the same level (20 ng mL^−1^). After the SPE procedure, the eluates were evaporated to dryness separately, and then separately reconstituted into 300 µL of the MeOH:H_2_O (50:50, *v*/*v*) mixture, and next both analyzed by HPLC-FLD.

**Figure 4:**
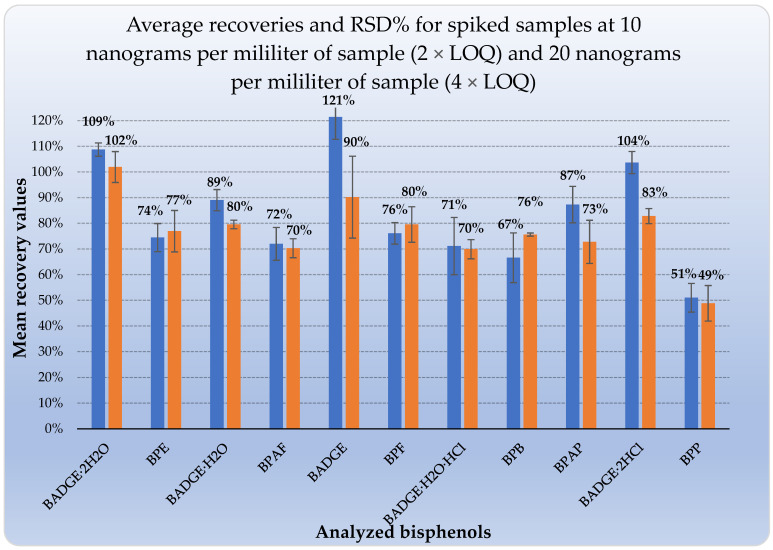
Mean recoveries (%) and relative standard deviations expressed as a percentage (RSD%) for mixture of bisphenols extracted by SPE using an Oasis HLB SPE column.

From the eleven bisphenols, the majority of the analytes showed satisfactory average recoveries (Figure 4) for both concentrations levels of 10 ng mL^−1^ (2 × LOQ) and 20 ng mL^−1^ (4 × LOQ), ranging between 67% and 121% for 2 × LOQ (Figure 4, marked pomegranate) and ranging between 70% to 102% for 4 × LOQ (Figure 4, marked orange). Only BPP (with the highest of log *p* value) demonstrated the lowest recovery values of 51% and 49% for 2 × LOQ and 4 × LOQ, respectively.

Another important aspect considered in this study was the possible presence of matrix effects that can be a source of serious problems in correct quantification. Studies performed revelated that considerable overestimations of concentrations could be observed if calibration curves were obtained with the standards prepared in a pure solvent, making it necessary to use spiked amniotic fluid samples as calibration standards for reliable quantifications. Details of the validation studies of the analytical methods can be obtained from References [17,18] and the Materials and Methods section (2.5.4. Matrix Effect—Assessment of the Degree of Matrix Interference). A matrix effect can be defined as a direct or indirect alteration or interference in response due to the presence of unintended analytes (for analysis) or other interfering substances in the sample. Due to this phenomenon, strong signal enhancement is observed when the analyte and co-eluting compound(s) have the same retention time. Generally, signal suppression occurs because other compounds interfere with the detection of the peak of interest. This results in a reduction in the quantum yield since some of the excitation is absorbed by the interferences or the emission is scattered or absorb by other interferences.

For that reason, in this manuscript the matrix effect is expressed as the percentage difference in a signal from the bisphenol (analyte) in the amniotic fluid sample (spiked matrix) compared to the signal in a pure solvent, as presented in Table 3.

The target compounds may be affected with the matrix co-elution, resulting in ion suppression or enhancement. We evaluated the slopes of the matrix-matched calibration curves to those of solvent-based calibration curves to examine the matrix effects. Matrix effects can be ignored if the slope ratios of the matrix and solvent are between 0.9 and 1.1, but they can be interpreted as enhancing the matrix with values more than 1.1 and suppressing the matrix with values lower than 0.9 in this experiment. Pairs of slope ratios (matrix (amniotic fluid)—solvent) were investigated and compared for eleven determined bisphenols.

The aim of this study was to develop a method that provides optimal recovery values for seven selected bisphenols while maintaining adequate purification of samples and a low matrix effect.

After the optimization procedure with application of solid-phase extraction on Oasis HLB SPE columns, some of bisphenols were determined with no significant or small matrix effects (Table 4). After optimization of the SPE procedure, a total of 20 samples were evaluated regarding the matrix effect. After their study were obtained the following ranges of influence of the matrix effect for all analytes (Table 4): none (from 0% to 10%); three (from 11% to 20%); three (from 21% to 30%); none (from 31% to 40%); three (above 40% to 55%); one (from 0% to −10%); none (from −11% to −20%); and one (from −21% to −30%).

Replicated measurements were used to assess the accuracy of the results (one sample was analyzed six times during next three days (total 18 times)). Average recovery values and RSD% (inter-day repeatability (*n* = 18)) for the majority bisphenols were in the ranges of 66.6–121.6% and 1.5–16.4%, respectively (Table 5). The intra-laboratory reproducibility also was evaluated. Average recovery values and RSD% (two analysts; *n* = 12) for the majority of bisphenols were in the ranges of 67.8–121.3% and 1.1–16.3%, respectively (Table 5). These results met our requirements for repeatability and inter- and intra-laboratory reproducibility [17,18].

The validated method was applied to the analysis of bisphenols in amniotic fluid collected from women during amniocentesis. The samples were analyzed utilizing extraction and chromatographic conditions proposed by authors in this paper. Successful purification by acetonitrile extraction, as well as the presence of enrichment steps in the extraction SPE procedure, allows determination of bisphenols at a low concentration (nanogram per milliliter of amniotic fluid). Residues of four analytes (BADGE·2H_2_O, BPAF, BADGE and BADGE·2HCl) were detected (below LOQ) in amniotic fluid collected from women during amniocentesis (Table 6).

In another HPLC-MS/MS study also, prior to maternal administration of bisphenols, the BPA, BPS and BPF concentrations measured in fetal amniotic fluid of pregnant sheep were < LOQ (<0.33 ± 0.0), 0.3 ± 0.2 and <LOQ (<0.5 ± 0.0) ng mL^−1^, respectively [3].

**Figure 5:**
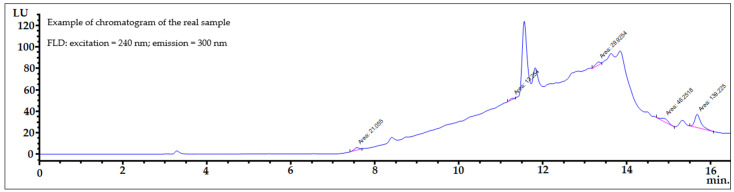
HPLC-FLD chromatogram of a human amniotic fluid sample with the detected five bisphenol residues (integrated peaks).

A series of experiments were conducted applying a sensitive HPLC-FLD system, which is less expensive than often used liquid chromatography coupled with other detection techniques such as mass spectrometry (LC-MS) or tandem mass spectrometry (LC-MS/MS). As shown in Figure 5, identification of bisphenol residues in amniotic fluid samples confirms the usefulness of the elaborated analytical procedure with application of sensitive fluorescence detection (FLD). Compared to the earlier procedures described by the authors [14,19], the advantage of this procedure is the identification of bisphenols in amniotic fluid samples (without the need to combine evaporated eluates from several samples). The conditions of the chromatographic analysis and the parameters of the FLD detector were optimized. Sufficient sensitivity HPLC-FLD was achieved applying reinforcements of the described studies to analytes (14 from range 1–18). The optimized conditions of the analyses allow for the selective enhancement of analytes in amniotic fluid samples, which are enough separated from the remaining matrix interferences. During the HPLC-FLD experiments a moderate reinforcement (14) of studies analytes from the applicable reinforcement range (1–18) was used. Obviously, after application of the higher levels of the analytes’ reinforcement (e.g., 15–18), there will be a possibility of quantification of much lower concentrations of bisphenols. The results demonstrated clearly that the approach developed provides reliable, simple, rapid and environmentally friendly quantification and identification of eleven bisphenols in a very rarely studied matrix and could be used for biomonitoring bisphenols in amniotic fluid collected from women during amniocentesis.

## 4. Discussion

Most of the described analytical methods [13,14,19,20,21,22,23,24,25,26,27,28,29,30,31,32,33] are based on the use of high-performance liquid chromatography coupled with mass spectrometry (HPLC-MS) or tandem mass spectrometry (HPLC-MS/MS) [13,20,27,28,29,30], ultra-performance liquid chromatography coupled with tandem mass spectrometry [31], as well as gas chromatography coupled with mass spectrometry (GC-MS) or tandem mass spectrometry (GS-MS/MS) [22,23,24,25,26,32]. Previous studies detected bisphenols in amniotic fluid, follicular fluid, placental tissue, sperm, cord blood, fetal serum, adipose tissue [26,27,28,29,32,33], urine or peripheral blood samples [27], and human breast milk samples [13,14,26,30].

In another study it has been determined the BPA exposure levels in various bodily fluids and tissues of pregnant women, describing fetus and infant exposures to BPA based on associations and BPA ratios in mother–neonate paired samples [26]. Maternal serum, urine, placenta, breast milk, cord serum and neonatal urine samples were collected from 318 mother–neonate pairs at six university hospitals in Korea. BPA levels were detected using liquid chromatography tandem mass spectrometry. BPA was detected in 79.5–100% of the maternal and fetal samples. The median BPA concentration in the samples decreased in the order of neonatal urine (4.75 ng mL^−1^), maternal urine (2.86 ng mL^−1^), cord serum (1.71 ng mL^−1^), maternal serum (1.56 ng mL^−1^), breast milk (0.74 ng mL^−1^) and the placenta (0.53 ng g^−1^) [26].

Because gestational BPS can disrupt placental function and result in reproductive and metabolic disorders in the progeny, the aim of the study described by Veiga-Lopez et al. was to investigate BPS and BPF toxicokinetics during pregnancy using an in vivo approach [3]. Described by [3], fetal catheterizations were conducted in pregnant sheep (*n* = 6) at mid-pregnancy and injected with either a single dose of BPS (*n* = 3, 0.5 mg/kg, s.c.), or a combination of BPS, BPF and BPA (*n* = 3, 0.5 mg/kg for each chemical, s.c.). Maternal and fetal blood and urine and amniotic fluid samples were collected over 72 h and analyzed for bisphenols by HPLC-MS/MS [3]. Veiga-Lopez et al. observed significant differences in the half-life, maximum concentration and total body clearance in maternal circulation among bisphenols. Longer half-lives were observed in fetal vs. maternal circulation for all bisphenols. Fetal toxicokinetics differed among bisphenols with BPSc having the longest fetal half-life. All bisphenols reached basal levels at 48 h in maternal plasma, but were still detectable in amniotic fluid, fetal urine and fetal plasma at 72 h [3].

Yi et al. [28] analyzed BPA with LC/MS/MS and HPLC/FLD in human breast milk and conducted a comparison of two methods in the analyzed BPA levels. The limits of quantification (LOQs) obtained for bisphenols were similar in the two methods, i.e., 1.8 and 1.3 ng mL^−1^ for the HPLC/FLD and LC/MS/MS assays, respectively [28]. In addition, the detection range of BPA was broader in the HPLC method than the LC/MS/MS method [28].

Tuzimski and Szubartowski have described the optimization of the conditions for the identification and quantitative analysis of bisphenols by HPLC-FLD and the optimization of the extraction conditions of analytes in urine samples using the SPE technique with Strata Phenyl SPE columns [19]. The authors proposed a sensitive, cost-effective and simple high-performance liquid chromatography method with fluorescence detection (HPLC-FLD) for the simultaneous determination of the three bisphenols such as bisphenol A bis (2,3-dihydroxypropyl), ether (BADGE 2H_2_O), bisphenol F (BPF) and bisphenol E (BPE) in human urine samples. During HPLC-FLD analysis, where from 6 min reinforcements to 10 or 12 were used, bisphenols were identified. Optimizing the conditions of the chromatographic analysis, the parameters of the FLD detector, including the use of an appropriate (optimal) amplification of the analytes (10 or 12) from the sixth minute of the chromatographic process, allowed for the selective enhancement of bisphenols in urine samples [19].

Fetal serum, cord blood and meconium are all appropriate matrices for monitoring fetal environmental exposures. However, only amniotic fluid (AF) and certain surrogate matrices (i.e., maternal serum, plasma, urine or placental tissue) can provide information prior to delivery to inform intervention strategies directed at improving perinatal outcomes. Amniotic fluid (AF) is a biological medium uniquely suited for the study of early exposure of the human fetus to environmental contaminants acquired by the mother before and during pregnancy.

The indication for amniocentesis was confirmation or exclusion of genetic aberrations such as trisomy 13 (Patau’s syndrome), trisomy 18 (Edwards syndrome) and trisomy 21 (Down syndrome), and on heritable diseases in high-risk pregnancies as well as the possibility of preterm birth. Four of the twenty women had pregnancy complications (fetal death during previous pregnancy). Newborns in these patients were born earlier and the presence of some bisphenols in the in amniotic fluid samples was initially identified.

A statistical study will be performed after additional confirmation of the identity of the analytes in the biological samples by tandem mass spectrometry. At this stage of the experiments, our main goal was to propose an extraction technique and analytical method for the identification and quantification of bisphenols in amniotic fluid samples. The statistical study and the evaluation and interpretation of the obtained research results is currently underway, and we hope it can be published in the future.

## 5. Conclusions

In summary, amniotic fluid (AF) is an information-rich bodily fluid of diagnostic value with untapped potential. It can serve to determine and quantify toxic environmental exposures to the fetus and may aid in elucidation of causes of the high incidence of preterm birth.

To date, no modern procedures have been developed that would enable the simultaneous identification and quantification of selected bisphenols in amniotic fluid samples at nanogram concentrations per milliliter of sample.

A specific, precise and accurate high-performance liquid chromatographic (HPLC) analytical method with a fluorescence detector (FLD) was established for the simultaneous determination of eleven bisphenols in amniotic fluid samples.

Under the optimized conditions, good linearity was obtained for the eleven bisphenols and the correlation coefficients (R^2^) ranged from 0.9866 to 0.9986. Recovery values for the ten bisphenols in spiked samples were 67.3–121.4% with intra-day and inter-day relative standard deviations (RSDs) from 1.3 to 16.4% (*n* = 30; apart from BPP).

In comparison to other chromatographic methods coupled with tandem mass spectrometry, the proposed HPLC-FLD method is a sensitive alternative for simultaneous quantitative analysis of bisphenols in amniotic fluid samples. Residues of five analytes (BADGE·2H_2_O, BPAF, BADGE, BADGE·H_2_O·HCl and BADGE·2HCl) were detected in amniotic fluid collected from women during amniocentesis.

The proposed SPE and HPLC-FLD procedure could be recommended for further effective and reliable analysis of selected bisphenols in small amounts of human amniotic fluid samples.

## Figures and Tables

**Table 1:** Characteristics of the patients who have undergone amniocentesis.

Patient Number	Age	The Indication for Amniocentesis
1	38	The risk of trisomy 13—1:120 The risk of trisomy 18—1:45
2	36	The risk of trisomy 13—1:12 The risk of trisomy 18—1:4 The risk of trisomy 21—1:4
3	30	The risk of trisomy 21—1:125
4	26	The risk of trisomy 13—1:79 Increased risk of trisomy 18
5	37	The risk of trisomy 18—1:118
6	38	The risk of trisomy 21—1:155
7	39	The risk of trisomy 13—1:252
8	24	The risk of trisomy 13—1:384 The risk of trisomy 18—1:268 The risk of trisomy 21—>1:4
9	39	The risk of trisomy 13—1:200 The risk of trisomy 21—1:119
10	32	The risk of trisomy 13—>1:50 The risk of trisomy 18—>1:50
11	41	Increased risk of trisomy 21
12	25	Increased risk of trisomy 21
13	28	The risk of trisomy 21—1:101
14	36	The risk of trisomy 21—1:300
15	29	The risk of trisomy 13—1:12 The risk of trisomy 18—1:470
16	30	Toxoplasmosis
17	31	Toxoplasmosis
18	30	Toxoplasmosis
19	44	Toxoplasmosis
20	36	Avidity of antibodies

**Table 2:** List of bisphenols used in this study and their physicochemical properties.

No.	Bisphenol	*IUPAC* Name	Chemical Structure	Molecular Weight	Log *P*	*H* Donors	*H* Acceptors	
1	BADGE∙2H_2_O	3-[4-[2-[4-(2,3-dihydroxypropoxy)phenyl]propan-2-yl]phenoxy]propane-1,2-diol	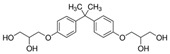	376.4	2.1	4	6	Mixture 1
2	BPE	4-[1-(4-hydroxyphenyl)ethyl]phenol	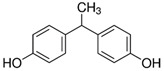	214.3	3.9	2	2	
3	BADGE∙H_2_O	3-[4-[2-[4-(oxiran-2-ylmethoxy)phenyl]propan-2-yl]phenoxy]propane-1,2-diol	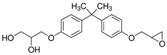	358.4	3.1	2	5	
4	BPAF	4-[1,1,1,3,3,3-hexafluoro-2-(4-hydroxyphenyl)propan-2-yl]phenol	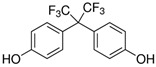	336.2	4.5	2	8	
5	BADGE	2-[[4-[2-[4-(oxiran-2-ylmethoxy)phenyl]propan-2-yl]phenoxy]methyl]oxirane	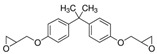	340.4	4.0	0	4	
6	BPF	4-[(4-hydroxyphenyl)methyl]phenol	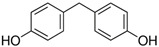	200.2	2.9	2	2	Mixture 2
7	BADGE∙H_2_O∙HCl	3-[4-[2-[4-(3-chloro-2-hydroxypropoxy)phenyl]propan-2-yl]phenoxy]propane-1,2-diol	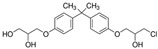	394.9	3.3	3	5	
8	BPB	4-[2-(4-hydroxyphenyl)butan-2-yl]phenol	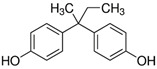	242.3	3.9	2	2	
9	BPAP	4-[1-(4-hydroxyphenyl)-1-phenylethyl]phenol	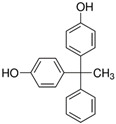	290.4	4.4	2	2	
10	BADGE∙2HCl	1-chloro-3-[4-[2-[4-(3-chloro-2-hydroxypropoxy)phenyl]propan-2-yl]phenoxy]propan-2-ol	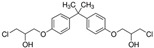	413.3	4.6	2	4	
11	BPP	4-[2-[4-[2-(4-hydroxyphenyl)propan-2-yl]phenyl]propan-2-yl]phenol	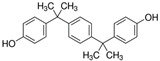	346.5	6.1	2	2	

**Table 3:** Validation parameters for the method: retention times, calibration curves equations (which were constructed using methanol), correlation coefficients (R^2^), limits of detection (LODs) and limits of quantification (LOQs) obtained for the 11 bisphenols by HPLC-FLD.

No.	Bisphenol	Retention Time (t_r_), min.	Concentration Range, ng mL^−1^	Linear Regression	Coefficient of Determination (R^2^)	Limit of Detection (LOD), ng mL^−1^	Limit of Quantification (LOQ), ng mL^−1^
1	BADGE∙2H_2_O	~7.5	0.5–20	*y* = 10.969*x* − 3.5584	0.9954	1.3	4.0
2	BPE	~10.8	0.5–20	*y* = 9.1235*x* + 2.9894	0.9976	1.0	2.9
3	BADGE∙H_2_O	~11.6	0.5–20	*y* = 10.21*x* + 14.406	0.9966	1.1	3.5
4	BPAF	~13.2	0.5–20	*y* = 13.873*x* − 8.1584	0.9971	1.1	3.2
5	BADGE	~15.7	0.5–20	*y* = 9.3354*x* + 15.447	0.9866	2.3	6.9
6	BPF	~10.1	0.5–20	*y* = 8.1827*x* − 8.6264	0.9954	1.3	4.1
7	BADGE∙H_2_O∙HCl	~11.3	0.5–20	*y* = 12.93*x* − 3.0183	0.9986	0.8	2.4
8	BPB	~12.6	0.5–20	*y* = 8.1644*x* − 3.086	0.9912	1.9	5.6
9	BPAP	~13.3	0.5–20	*y* = 10.258*x* − 13.628	0.9983	2.5	7.5
10	BADGE∙2HCl	~14.9	0.5–20	*y* = 13.658*x* − 5.6061	0.9922	1.7	5.3
11	BPP	~15.9	0.5–20	*y* = 15.556*x* − 15.54	0.9869	2.3	5.9

**Table 4:** Validation parameters for the method: retention times, calibration curves equations (which were constructed using the averaged matrix of amniotic fluid), correlation coefficients (R^2^), limits of detection (LODs), limits of quantification (LOQs) and matrix effect (ME) obtained for the 11 bisphenols by HPLC-FLD.

No.	Bisphenol	Retention Time (t_r_), min.	Concentration Range, ng/mL	Linear Regression	Coefficient of Determination (R^2^)	Limit of Detection (LOD), ng/mL	Limit of Quantification (LOQ) ng/mL	Matrix Effect (ME)
1	BADGE∙2H_2_O	~7.5	2.5–50	*y* = 10.317*x* + 82.514	0.9973	2.5	7.7	29%
2	BPE	~10.8	2.5–50	*y* = 6.9794*x* + 9.4408	0.9995	1.1	3.2	−25%
3	BADGE∙H_2_O	~11.6	2.5–50	*y* = 9.6941*x* + 1050.9	0.9982	2.1	6.3	50%
4	BPAF	~13.2	2.5–50	*y* = 11.84*x* + 33.191	0.9992	1.4	4.2	11%
5	BADGE	~15.7	2.5–50	*y* = 7.9407*x* + 96.777	0.9888	5.2	15.7	13%
6	BPF	~10.1	2.5–50	*y* = 7.6505*x* + 19.466	0.9927	4.2	12.6	19%
7	BADGE∙H_2_O∙ HCl	~11.3	2.5–50	*y* = 8.8985*x* + 5.7375	0.9969	5.2	15.6	−6%
8	BPB	~12.6	2.5–50	*y* = 8.8985*x* + 5.7375	0.9969	2.7	8.2	22%
9	BPAP	~13.3	2.5–50	*y* = 11.913*x* + 38.88	0.9957	3.2	9.7	55%
10	BADGE∙2HCl	~14.9	2.5–50	*y* = 13.312*x* + 43.256	0.9958	3.2	9.6	23%
11	BPP	~15.9	2.5–50	*y* = 16.466*x* + 129.07	0.9987	1.8	5.4	50%

**Table 5:** Intra- and inter-day data studied for the proposed HPLC-FLD method for the analysis of 11 bisphenols in spiked human amniotic fluid samples at 10 ng mL^−1^ and 20 ng mL^−1^ after SPE procedure.

**Recoveries Obtained for Fortification Level at 10 ng per Mililiter of Sample (2 × LOQ) after Procedure Shown in Figure 1**																
**Bisphenol**	**Intra-Day Repeatability ^a^**						**Inter-Day Repeatability ^b^** **(*n* = 18)**		**Intra-Laboratory Reproducibility ^c^**						**Overall ^d^** **(*n* = 30)**	
**Name**	**Day 1 (*n* = 6)**		**Day 2 (*n* = 6)**		**Day 3 (*n* = 6)**				**Analyst 1** **(*n* = 6)**		**Analyst 2** **(*n* = 6)**		**Mean (*n* = 12)**			
	**Rec.^e^**	**RSD%**	**Rec.^e^**	**RSD%**	**Rec.^e^**	**RSD%**	**Rec.^e^**	**RSD%**	**Rec.^e^**	**RSD%**	**Rec.^e^**	**RSD%**	**Rec.^e^**	**RSD%**	**Rec.^e^**	**RSD%**
BADGE∙2H_2_O	109.2	2.7	110.7	4.3	110.7	2.3	110.2	3.1	108.7	4.1	107.3	3.3	108.0	3.7	109.2	3.4
BPE	74.8	6.6	76.2	4.5	76.0	6.3	75.7	5.8	76.2	5.2	73.3	4.9	74.8	5.1	75.3	5.4
BADGE∙H_2_O	89.3	3.9	87.8	5.7	89.0	3.6	88.7	4.4	89.7	4.4	88.5	4.4	89.1	4.4	89.0	4.4
BPAF	71.7	5.4	72.7	6.1	71.8	5.8	72.1	5.8	72.8	5.7	72.2	8.4	72.5	7.1	72.5	6.4
BADGE	121.2	8.6	122.2	9.3	121.3	9.9	121.6	9.3	121.3	10.6	121.3	8.5	121.3	9.5	121.4	9.4
BPF	76.3	4.9	76.7	4.7	75.7	3.9	76.2	4.5	75.2	4.6	74.8	4.0	75.0	4.3	75.6	4.4
BADGE∙H_2_O∙HCl	70.3	9.6	71.8	11.7	71.8	10.7	71.3	10.6	71.0	11.9	70.8	11.4	70.9	11.6	71.3	11.1
BPB	66.2	10.7	66.8	10.1	66.8	9.4	66.6	10.1	67.7	9.5	67.8	11.3	67.8	10.4	67.3	10.2
BPAP	86.8	7.7	87.7	7.9	86.7	7.8	87.1	7.8	86.2	6.3	86.8	7.5	86.5	6.9	87.0	7.4
BADGE∙2HCl	104.5	3.8	104.0	4.9	104.7	4.2	104.4	4.3	104.3	3.2	104.3	4.4	104.3	3.8	104.4	4.1
BPP	51.3	6.1	50.8	6.2	51.7	6.9	51.3	6.4	50.8	7.0	51.8	4.9	51.3	6.0	51.3	6.2
**Recoveries Obtained for Fortification Level at 20 ng per Mililiter of Sample (4 × LOQ) after Procedure Shown in Figure 1**																
**Bisphenol**	**Intra-Day Repeatability ^a^**						**Inter-Day Repeatability ^b^** **(*n* = 18)**		**Intra-Laboratory Reproducibility ^c^**						**Overall ^d^** **(*n* = 30)**	
**Name**	**Day 1 (*n* = 6)**		**Day 2 (*n* = 6)**		**Day 3 (*n* = 6)**				**Analyst 1** **(*n* = 6)**		**Analyst 2** **(*n* = 6)**		**Mean (*n* = 12)**			
	**Rec.^e^**	**RSD%**	**Rec.^e^**	**RSD%**	**Rec.^e^**	**RSD%**	**Rec.^e^**	**RSD%**	**Rec.^e^**	**RSD%**	**Rec.^e^**	**RSD%**	**Rec.^e^**	**RSD%**	**Rec.^e^**	**RSD%**
BADGE∙2H_2_O	102.2	6.5	102.5	6.2	101.8	4.9	102.2	5.8	101.3	6.7	101.5	6.6	101.4	6.6	101.8	6.2
BPE	77.8	8.9	77.2	7.5	77.3	8.5	77.4	8.3	76.5	7.2	77.2	7.2	76.8	7.2	77.1	7.7
BADGE∙H_2_O	80.8	2.3	80.7	1.8	81.2	3.2	80.9	2.5	80.5	2.7	79.7	2.0	80.1	2.3	80.5	2.4
BPAF	70.2	3.9	71.0	4.7	69.8	4.2	70.3	4.3	71.2	4.8	70.5	3.1	70.8	4.0	70.6	4.1
BADGE	89.8	16.4	90.5	17.9	91.3	14.9	90.6	16.4	89.7	16.5	88.8	16.2	89.3	16.3	89.9	16.4
BPF	79.2	7.5	80.7	7.1	81.5	7.3	80.4	7.3	80.8	8.2	79.8	6.8	80.3	7.5	80.4	7.4
BADGE∙H_2_O∙HCl	69.8	3.5	70.2	3.8	71.0	5.1	70.3	4.1	69.7	3.8	71.3	5.5	70.5	4.6	70.4	4.4
BPB	75.3	1.3	75.5	1.5	75.0	1.9	75.3	1.5	76.7	1.2	76.5	1.0	76.6	1.1	75.9	1.3
BPAP	74.2	8.2	73.2	8.4	74.3	8.1	73.9	8.2	72.3	7.8	71.5	8.3	71.9	8.1	72.9	8.1
BADGE∙2HCl	82.5	3.8	83.2	3.6	83.5	2.9	83.1	3.4	83.7	3.6	82.8	2.9	83.3	3.2	83.2	3.3
BPP	49.7	7.3	49.3	7.8	49.8	4.7	49.6	6.6	48.7	8.0	47.7	7.7	48.2	7.9	48.9	7.2

^a^—mean recovery% and RSD% for within-day results of batch of six samples per day (*n* = 6). ^b^—mean recovery% and RSD% from 18 samples analyzed in three different days (*n* = 6 for each day). ^c^—mean recovery% and RSD% from experiments conducted by two different analysts (*n* = 6 for each operator) and average results (*n* = 12). ^d^—average recovery% and RSD% from all experiments (*n* = 30). ^e^—Recovery (%).

**Table 6:** Results of the determination of BPs in both different human amniotic fluid samples.

Bisphenol	Sample 1	Sample 2
BADGE∙2H_2_O	<LOQ	<LOQ
BPE	not detected	not detected
BADGE∙H_2_O	not detected	not detected
BPAF	<LOQ	<LOQ
BADGE	<LOQ	<LOQ
BPF	not detected	not detected
BADGE∙H_2_O∙HCl	<LOQ	not detected
BPB	not detected	not detected
BPAP	not detected	not detected
BADGE∙2HCl	<LOQ	<LOQ
BPP	not detected	not detected

<LOQ—below limit of quantification.

## Data Availability

Data are contained within the article.
